# Pentachloronitrobenzene Reduces the Proliferative Capacity of Zebrafish Embryonic Cardiomyocytes via Oxidative Stress

**DOI:** 10.3390/toxics10060299

**Published:** 2022-06-01

**Authors:** Guoqiang Fan, Tianzhu Shen, Kun Jia, Xiaoping Xiao, Zhanfeng Wu, Fanghua Gong, Huiqiang Lu

**Affiliations:** 1 School of Pharmacy, Wenzhou Medical University, Wenzhou 325000, China; 864473680@wmu.edu.cn (G.F.); stz960506@163.com (T.S.); 2 Center for Drug Screening and Research, School of Geography and Environmental Engineering, Gannan Normal University, Ganzhou 341000, China; 402432419005@email.ncu.edu.cn (K.J.); 1400063@gnnu.edu.cn (X.X.); 3 Institute of Chemistry, Chinese Academy of Sciences, Beijing 100000, China; wuzhanfeng@iccas.ac.cn; 4 Center for Developmental Biology of Jinggangshan University, College of Life Sciences, Jinggangshan University, Ji’an 343009, China

**Keywords:** pentachloronitrobenzene, pericardial edema, reactive oxygen species, proliferation of cardiomyocytes, zebrafish

## Abstract

Pentachloronitrobenzene (PCNB) is an organochlorine protective fungicide mainly used as a soil and seed fungicide. Currently, there are few reports on the toxicity of PCNB to zebrafish embryo. Here, we evaluated the toxicity of PCNB in aquatic vertebrates using a zebrafish model. Exposure of zebrafish embryos to PCNB at concentrations of 0.25 mg/L, 0.5 mg/L, and 0.75 mg/L from 6 hpf to 72 hpf resulted in abnormal embryonic development, including cardiac malformation, pericardial edema, decreased heart rate, decreased blood flow velocity, deposition at yolk sac, shortened body length, and increased distance between venous sinus and arterial bulb (SV-BA). The expression of genes related to cardiac development was disordered. However, due to the unstable embryo status in the 0.75 mg/L exposure concentration group, the effect of PCNB on the expression levels of cardiac-related genes was not concentration-dependent. We found that PCNB increased reactive oxygen species stress levels in zebrafish, increased malondialdehyde (MDA) content and catalase (CAT) activity, and decreased superoxide dismutase (SOD) activity. The increased level of oxidative stress reduced the proliferation ability of zebrafish cardiomyocytes, and the expressions of zebrafish proliferation-related genes such as *cdk-2*, *cdk-6*, *ccnd1*, and *ccne1* were significantly down-regulated. Astaxanthin (AST) attenuates PCNB-induced reduction in zebrafish cardiomyocyte proliferation by reducing oxidative stress levels. Our study shows that PCNB can cause severe oxidative stress in zebrafish, thereby reducing the proliferative capacity of cardiomyocytes, resulting in zebrafish cardiotoxicity.

## 1. Introduction

Pentachloronitrobenzene (PCNB) is an organochlorine protective fungicide which is primarily used for the prevention and control of vegetable seedling diseases, such as blight [1], damping-off [2], and anthracnose. However, due to its chemical stability, it can effectively persist in soil for 78.5 days and in water even up to 1042 days [3]. In the past few decades, PCNB has been extensively used for agriculture purposes, and in China, the usage of PCNB has even reached 1000 t a^−1^ [4]. It has been established that because of its wide availability and chemical stability, it can accumulate in large amounts in soil, water and even in the air [5]. PCNB has been detected in various vegetables [6], soils [6,7], surface water [8] and traditional Chinese medicine [9]. In recent years, since many countries have begun to restrict the use of PCNB, few articles have reported the concentration of PCNB in the environment, but in China and some other countries, PCNB is still abused, and PCNB can even be detected in some Chinese medicinal materials. For example, the concentration of PCNB in soil cultivating ginseng was 0.2 mg/kg and the PCNB level in the ginseng reached 1 mg/kg [10]. Bioaccumulation of PCNB has been detected even in some aquatic organisms such as the golden trout, black trout, and rainbow trout [11]. According to the current reports, PCNB can directly damage the human intestinal epithelial cells [12], and cause serious skin as well as eye irritation [13]. On 27 October 2017, PCNB was listed as a carcinogen by the World Health Organization’s International Agency for Research on Cancer. At present, studies related to the cardiotoxicity of PCNB have not been reported, and there are only few studies describing the potential toxicity of PCNB in aquatic organisms. To this end, we have used zebrafish as a model to investigate the toxicity of PCNB to embryonic development and cardiac toxicity.

Zebrafish has approximately 87% gene homology with humans. Its signal transduction pathway is similar to that of humans, and its biological structure and physiological functions are identical to that of mammals. It is a small organism [14] has a short development cycle, requires only a short experimental process [15], low cost, can be developed externally, and is transparent (it can be used to directly observe the effect of the drugs on internal organs). Currently, zebrafish is widely used in developmental biology research, human disease model research, new drug screening, drug toxicity, cardiovascular research, safety evaluation, and environmental toxicology related research [14]. Moreover, compared with mice and chickens, zebrafish have been found to be more suitable for the study of cardiotoxicity, because zebrafish embryos can obtain oxygen through the process of passive diffusion at an early stage, which enables them to survive even with severe heart defects [16]. As an ideal animal model for cardiovascular research [17], zebrafish has been widely used in the screening of drug cardiotoxicity. For example, in 2020, Huang et al., studied the cardiotoxicity of the herbicide Oxadiazon-Butachlor [18] on zebrafish, and in 2021, Wan et al., analyzed the toxicity of the clinical drug Cyclosporine A [19] on zebrafish heart. As the first organ in zebrafish to develop and function [20], the heart begins to develop as early as at 24 h post-fertilization (hpf) [7]. It can effectively promote blood flow, provide oxygen and nutrients (such as water, inorganic salts, glucose, protein, various water-soluble vitamins, etc.) to the various organs, and can remove the various metabolic end-products. According to previous reports, the various marker genes for early heart development in zebrafish include *myh6* [21,22], *nppa* [23,24], *gata4* [17,25], *nkx2.5* [26,27], *tbx5a* [28], *tbx2b* [29], etc. Thus, with identification of these important marker genes of early heart development in zebrafish, it is easier for researchers to study the mechanisms of zebrafish cardiotoxicity.

It has been established that the zebrafish heart has a strong regenerative ability [30,31], which is different from mammals and this is largely due to the proliferation ability of cardiomyocytes [32]. The reduced proliferative capacity of cardiomyocytes is one of the main obstacles in the regeneration process after myocardial infarction [33], and appears to be inseparable from the regulation of cell cycle factors, such as *cdk-2*, *cdk-6*, *ccnd1*, *ccne1*, etc. [34,35].

Oxidative stress refers to the process in which the production of reactive oxygen species in the body exceeds the body’s ability to eliminate reactive oxygen species (ROS) upon exposure to harmful stimuli, and can serve as an important factor leading to aging and various chronic diseases [36]. According to current reports, the presence of oxidative stress can effectively lead to cardiotoxicity [22], neurotoxicity [37], apoptosis [38], cardiomyocyte cell cycle arrest [39], etc., in zebrafish. Astaxanthin (AST) is one of the most powerful antioxidants known to date. In toxicology experiments, AST was found to significantly alleviate and combat the negative effects of oxidative stress, and is often used to combat the toxicity caused by oxidative stress [40,41]. In 2020, the experimental results of Cao and Huang et al. showed that AST could alleviate the cardiotoxicity of zebrafish embryos by reducing their oxidative stress activity [18,42].

In this article, we treated zebrafish embryos with PCNB and thereafter analyzed the resulting morphological changes, cardiotoxicity and the level of oxidative stress through ROS staining. We also conducted real-time fluorescence quantitative PCR of the various heart-related and proliferation-related genes, and carried out immunofluorescence staining. Our study showed for the first time that PCNB was significantly cardiotoxic to zebrafish embryos and caused severe oxidative stress. The cardiotoxicity and oxidative stress induced by PCNB on zebrafish embryos could be effectively rescued by astaxanthin intervention. These findings can have important implications for assessing the potential toxicity of PCNB on both aquatic organisms and humans.

## 2. Materials and Methods

### 2.1. Zebrafish Strains and Rearing

This study used zebrafish (*Danio rerio*, zebrafish) as a model living creature for research. The experimental zebrafish strains used in the project were AB and *Tg (myl7: GFP)*. All zebrafish breeds were obtained from the National Zebrafish Resource Center and were raised under standard temperature (28 ± 1 °C) and 14 h light:10 h dark photoperiod conditions according to the Institutional Animal Care and Use Committee. Breeding zebrafish requires water with a pH of 7 and a conductivity of 500 μS/cm, feeding at 9:00 a.m. and 2:00 p.m. every day with brine shrimp larvae [19]. All zebrafish embryos were euthanized by ice bath after experimentation.

### 2.2. Experimental Reagents

The PCNB (CAS No.82-68-8 Analytical Standards, 99%) used in this study was procured from Aladdin Reagent Co., Ltd. The superoxide dismutase (SOD) kit (total measurement) (A001-1-1), catalase (CAT) visible light kit (A007-1-1), trace malondialdehyde (MDA) kit (A003-2-2) and the ROS assay kit (Code: E004-1-1, 100 T-500 T) were all from Nanjing Jiancheng Institute of Biological Engineering. The Ribonucleic Acid (RNA) extraction reagent TRIZOL (ET111-01) was obtained from Takara. The primary Proliferating Cell Nuclear Antigen (PCNA) antibody was obtained from Abcam UK. Perfect Start Green qPCR Super Mix kit (AQ601-04) and reverse transcriptase kit were obtained from Beijing Quanshijin Biotechnology Co., Ltd. (Beijing, China). Other biochemical reagents are analytical, purchased from Shenggong Biotechnology (Shanghai, China).

### 2.3. Embryo Collection

The night before, the male fish and female fish, in a ratio of 1:1, respectively, were placed into the mating tank and separated with a partition. The next morning the partition was removed, and the female fish laid their embryos under the impact of the male fish, and then fertilized naturally. Finally, the embryos were collected. A disposable pipette was used to suck out the dead, unfertilized embryos and other debris, and the remaining embryos were washed with pure water several times, placed in culture medium (a culture solution for zebrafish embryo culture, the culture solution composed of sodium chloride, potassium chloride, calcium chloride, magnesium sulfate, methylene blue and pure water, specific components and contents as follows: NaCl 17.5 g, KCl 0.75 g, CaCl_2_ 3 g, MgSO_4_ 2.5 g, methylene blue 0.5 mL, and pure water, making up 1000 mL.), and transferred to a constant temperature incubator at 28.5 °C [43]. The embryos used in the experiment were incubated with 0.003% (g/mL) N-phenylthiourea (PTU) after somito-genesis (12 hpf) and before the initiation of blood circulation (24 hpf). The production of melanin was inhibited for observation and recorded under the microscope.

### 2.4. Embryo Drug Treatment

According to the OECD principle, after observation and selection through the Leica stereo microscope, embryos with a development time of 6 h were selected. Next, 20 embryos were added to each well of the 6-well plate (The final solution volume per well is 8 mL), followed by the addition of 0.25 mg/L, 0.5 mg/L, and 0.75 mg/L of PCNB drug concentration for treatment. (The final exposure concentration is the concentration chosen based on the state in which the embryo appears phenotypic, and is also suitable for performing the experiment). The final exposure concentration was 0 mg/L, 0.25 mg/L, 0.5 mg/L and 0.75 mg/L. All selected exposure concentrations were lower than concentrations detected for PCNB in the environment. Since PCNB is difficult to dissolve in water, we dissolved it in dimethyl sulfoxide (DMSO) to prepare the stock solution of 10 mg/mL. The control group was treated with DMSO (according to the DMSO content of the 0.75 mg/L exposure concentration group as a control). The drug treatment was performed at 28.5 °C, the medium was changed and re-dosed every 24 hpf (PCNB is relatively constant during exposure and remains within the 10% range of nominal concentration (Figure S1, Table S2).), and the dead embryos were counted at 24 hpf, 48 hpf, and 72 hpf during the drug treatment. At 72 hpf, the number of heartbeats over 20 s was recorded. The embryos of different concentration treatment groups were selected and anesthetized with 0.15% tricaine and placed and set in 1% low-melting agarose. The most specific posture to show the phenotype was when the zebrafish’s eyes, body, and tail were in the same straight line—observation and shooting were carried out with a Leica microscope (Leica M205, Leica Microsystems Srl, Wetzlar, Germany).

### 2.5. Oxidative Stress Detection

At 72 hpf, we collected embryos at different exposure concentrations (about 15/tube) into 1.5 mL EP tubes and washed three times with phosphate buffered saline (PBS) for 5 min each. Next, we added 200 μL of DCFH-DA staining solution with a final concentration of 10 μM to each tube (the whole process was protected from light) for 30–60 min ROS staining, and the staining was terminated based on the staining situation [18]. A fluorescence microscope was used to observe and record the images. We used ImageJ software to quantify the fluorescence intensity of ROS staining results. The proteins were collected from the embryos with significant phenotypes, choosing the appropriate kits, and the concentration of malondialdehyde (MDA), catalase (CAT), and superoxide dismutase (SOD) was determined according to the operation steps given by the kit.

### 2.6. Paraffin Section and Hematoxylin-Eosin Staining (H & E) Staining

We collected 5–10 embryos at different exposure concentrations at 72 hpf. They were washed thrice with PBS for 5 min and kept in overnight incubation with 4% paraformaldehyde solution (PFA) at 4 °C. After dehydration with ethanol gradient and made transparent with xylene, the embryos were embedded in paraffin and sectioned by a Leica paraffin microtome, made into 5 μm sections. The tissue was collected on a glass slide and dried at 37 °C. After dewaxing with xylene and dehydration with ethanol, hematoxylin and eosin were stained according to the staining steps and timing in the literature [44,45], sealed with a neutral resin, covered with a glass cover, and dried for at least 8 h at 37 °C. The sections were observed and photographed with a microscope (Leica DM2500, Leica Microsystems Srl, Wetzlar, Germany).

### 2.7. Immunofluorescence Staining

After *Tg (myl7:GFP)* was drug-treated, 20 embryos were collected in each treatment group at 72 hpf, washed by PBS solution, and fixed with 4% PFA overnight. After dehydration with methanol, rehydration was carried out to better remove the pericardium. Then, immunofluorescence staining was performed with PCNA antibody at a 1:500 dilution, followed by overnight shaking at 4 °C as previously described [46]. After washing the embryos three times with PBS containing 0.1% Triton X-100, samples were incubated with 4′,6-diamidino-2-phenylindole (DAPI) dye for two hours at room temperature and washed again. Finally, these embryos were observed and photographed under a laser scanning confocal microscope (LeicaTCS SP8, Leica Microsystems Srl, Wetzlar, Germany).

### 2.8. Real-Time Fluorescence Quantitative PCR

At seventy-two hpf, each treatment group selected 30–40 embryos, and the TriZol kit was used to extract the total RNA from the embryo. (For reliability of the experimental results, the head and tail of zebrafish embryos were removed to ensure that the gene expression levels of zebrafish embryonic cardiomyocytes were detected as much as possible). Next, the Prime Script^®^RT kit was used to reverse the total RNA into complementary DNA [26,44]. We used the Applied Biosystems Step-One-plus Real-time PCR system (Applied Biosystem, Thermo Fisher, Waltham, CA, USA) to perform real-time fluorescent quantitative PCR; the samples were added according to the instructions of the Perfect Start Green qPCR Super Mix kit, and β-actin was used as an internal reference. The designed primers [22,26,45] were used for real-time fluorescent quantitative PCR. The experiment was repeated three times for each sample to improve the accuracy of the experiment and reduce errors. The primers used are described in Table S1.

### 2.9. Rescue Experiment

The embryos with a healthy condition at 6 hpf were selected, and we added 20 embryos per well and divided them into three experimental groups: Ctrl + DMSO, 0.5 mg/L PCNB, 0.5 mg/L PCNB + 30 nM AST. (Because AST was dissolved by DMSO, the same amount of DMSO was added to Ctrl as a reference). The culture medium was changed every 24 hpf. After 72 hpf, the phenotype was observed under a microscope and photographed.

### 2.10. Statistical Analysis

We used GraphPad Prism8 software to analyze the experimental data. Each data was repeated three times to reduce errors and contingency. Each time, we performed a normality analysis on all data before performing the analysis of significant differences, and all of the data presented a normal distribution. Thereby, a one-way ANOVA analysis of variance was used to compare the control group and the experimental group. The exact values of all *p*-values are shown in our paper.

## 3. Results

### 3.1. PCNB Causes Severe Damage to Zebrafish Embryos

**Figure 1:**
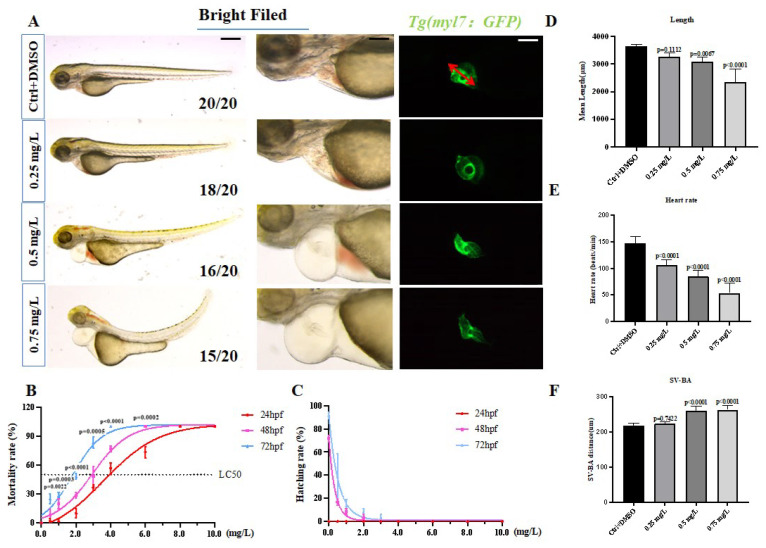
The phenotype of embryos treated with PBCN. (**A**). Bright field images of the whole body and heart and fluorescence microscope images of the heart. (**B**,**C**). Under different concentrations of PCNB, 24, 48, 72 hpf, the survival rate and hatching rate of embryos. (**D**–**F**). A statistical graph of the embryo’s body length, heart rate, and atrioventricular distance after 72 h of treatment with different concentrations. SV-BA refers to the distance from the atrium to the ventricle, the red arrow in the figure points. (*n* = 3, Means ± SD. Scale bar: 500 μm/100 μm).

Through the statistical analysis of mortality, we found that the mortality of embryos increased with an increase in the concentration of PCNB, and the lethal concentration was 50% (LC50) at 24, 48, and 72 hpf were 2, 3, and 4 mg/L, respectively (Figure 1B). Despite the concentration of 1 mg/L, the state of the zebrafish was not suitable for experimentation. In the end, we chose the concentration of 0.25, 0.5, 0.75 mg/L for treatment. After processing with the three concentrations of PCNB (0.25, 0.5, and 0.75 mg/L) for 72 h, we observed that pericardial edema, heart linearization, and yolk sac edema occurred in the high concentration group. There was even a severely curved trunk (Figure 1A), and brain hemorrhage and tail hemorrhage occurred in the middle and high concentration group (not explored in this article). The hatching rate also decreased along with an increased concentration (Figure 1C). We statically analyzed body length and found that this also decreased as the concentration increased (Figure 1D).

### 3.2. Cardiotoxicity of PCNB Treatment of Zebrafish

**Figure 2:**
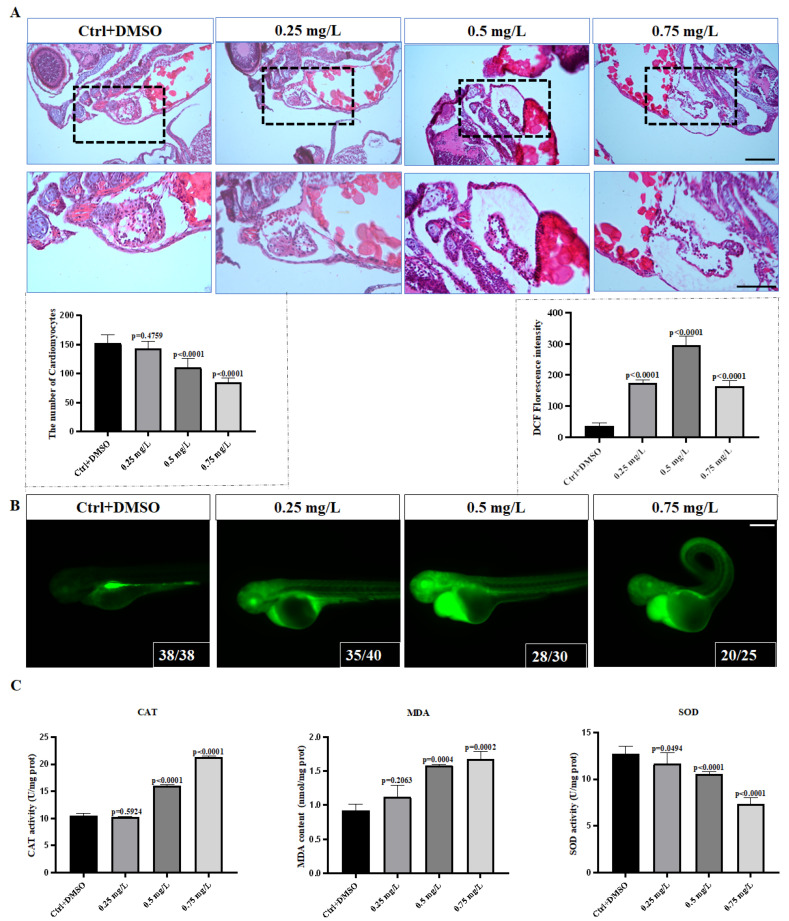
Hematoxylin-Eosin staining image and PCNB causes oxidative stress in the body. (**A**). Comparison of H&E staining in the control group and H&E staining in the PCNB treatment group. (**B**). Reactive oxygen species staining images of different treatment groups. (**C**). Malondialdehyde content, CAT activity, and SOD activity. Each difference is a comparison between the control group and the treatment group. (*n* = 3, Means ± SD. Scale bar: (**A**) 50 μm/25 μm. (**B**) 300 μm).

**Figure 3:**
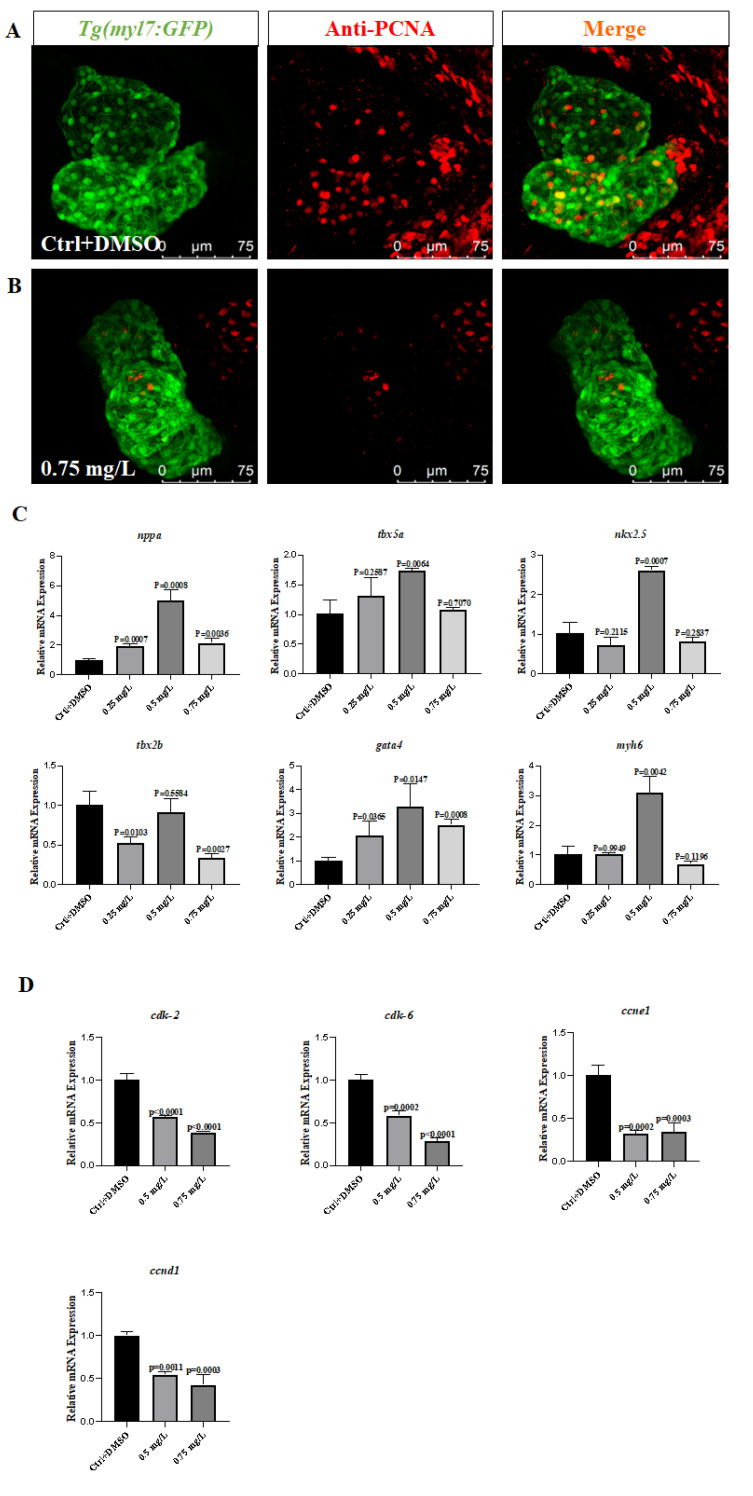
Pentachloronitrobenzene inhibited cardiac proliferation. (**A**). Cardiac proliferation in the Ctrl group (orange represents proliferating cells). (**B**). Cardiac proliferation in the 0.75 mg/L treatment group. (**C**). Expression levels of heart-related genes. (**D**). Cell proliferation-related gene expression. Each difference is a comparison between the control group and the treatment group. (*n* = 20, Means ± SD).

After we treated the *Tg (my17:GFP)* fish line with PCNB, we found that the heart of the middle and high concentration group was significantly linear, the distance between the atrium and the ventricle was enlarged, and there was a positive relationship with the concentration of PCNB (Figure 1F). The number of cardiomyocytes in the middle and high concentration group was significantly less than that in the control group (Figure 2A). We also counted the heart rate and counted the number of heartbeats for each sample over 20 s, and found that, as the concentration increased, the heart rate dropped significantly, reducing the pumping function of the heart (Figure 1E). Next, we performed Real-time fluorescence quantitative PCR on some specific expressed cardiac genes, such as *nkx2.5*, *nppa*, *myh6*, *gata4*, *tbx5a*, *tbx2b*, to further study the effect of PCNB on the zebrafish heart. We found that *tbx5a*, *nppa*, *myh6*, *gata4* were significantly upregulated, whereas *tbx2b* was significantly downregulated (Figure 3C). However, we found that the effect of PCNB on heart-related marker genes was not concentration-dependent, possibly because the embryo status of the 0.75 mg/L concentration group was unstable, so there were some disorder indicators.

### 3.3. PCNB Causes Severe Oxidative Stress in Zebrafish Embryos

Oxidative stress is an important factor in the onset of aging and disease, leading to inflammatory infiltration of neutrophils in the body, increased secretion of proteases, and production of large amounts of oxidative intermediate products, causing damage to the body. Superoxide dismutase (SOD), catalase (CAT), malondialdehyde (MDA), and reactive oxygen species (ROS) are used to indicate the degree of body oxidation. Reactive oxygen species staining in the embryos treated with PCNB showed that the oxidative stress in the body mainly concentrated on the pericardium and head (Figure 2B). The enzyme activity measurement of the PCNB treatment group showed that, with an increase in concentration, MDA content and CAT activity were significantly upregulated, and SOD activity was significantly reduced (Figure 2C), which implied that the body’s ability to scavenge oxygen free radicals was reduced, and the degree of peroxidation in the body was significantly increased, indicating that PCNB caused severe oxidative stress levels in zebrafish embryos, which may cause severe damage.

### 3.4. PCNB Reduces the Proliferation of Cardiomyocytes in Zebrafish Embryos

We observed the changes in proliferation in the heart using a laser confocal microscope via antibody coloration to verify whether the embryonic cardiomyocytes after treatment with PCNB are abnormal. The cardio-myocytes of *Tg (my17:GFP)* fish line have green fluorescence. Through the co-localization of cardiomyocytes and PCNA-marked proliferating cells, we found that embryos treated with 0.75 mg/L had a significant reduction in proliferating cells (Figure 3A,B). This showed that PCNB affected the normal proliferation of the embryonic heart, which made the heart develop abnormally. In addition, we performed Real-time fluorescence quantitative PCR to verify the changes in proliferation-related genes *cdk-2*, *cdk-6*, *ccnd1*, *ccne1*, and we found that *cdk-2*, *cdk-6*, *ccnd1*, *ccne1* were significantly reduced. This showed that PCNB lead to a decrease in the cardiomyocyte proliferation of zebrafish.

### 3.5. Astaxanthin Rescued the Oxidative Stress Damage Caused by PCNB

**Figure 4:**
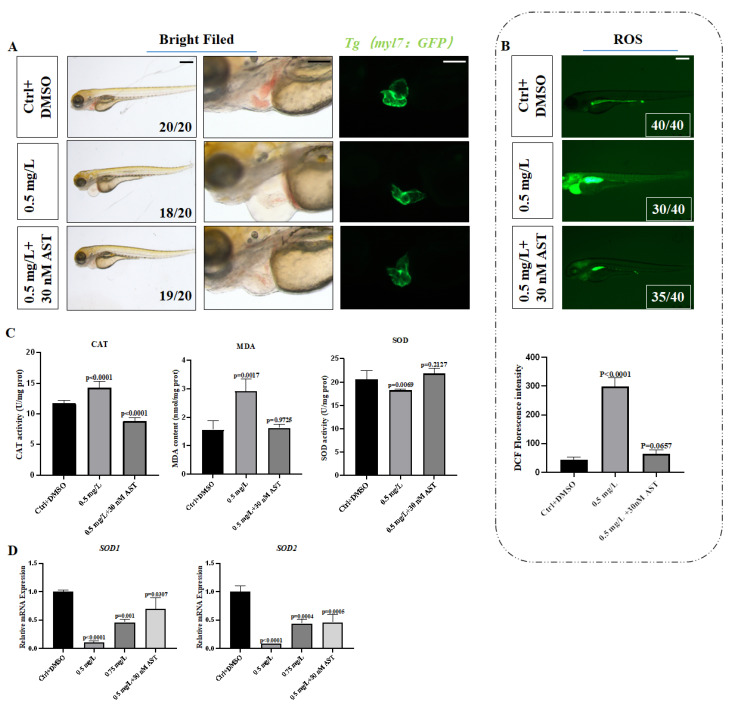
Astaxanthin rescued the oxidative stress caused by PCNB. (**A**). Bright field phenotypes and heart fluorescence images of the control group, medium concentration group, and AST rescue medium concentration group. (**B**). ROS-stained image of AST rescue. (**C**). MDA content, CAT activity, SOD activity. (**D**). Oxidative stress related gene expression level. Each difference is a comparison between the control group and the treatment group. (*n* = 3, Means ± SD. Scale bar: (**A**) 500 μm/100 μm. (**B**) 500 μm).

**Figure 5:**
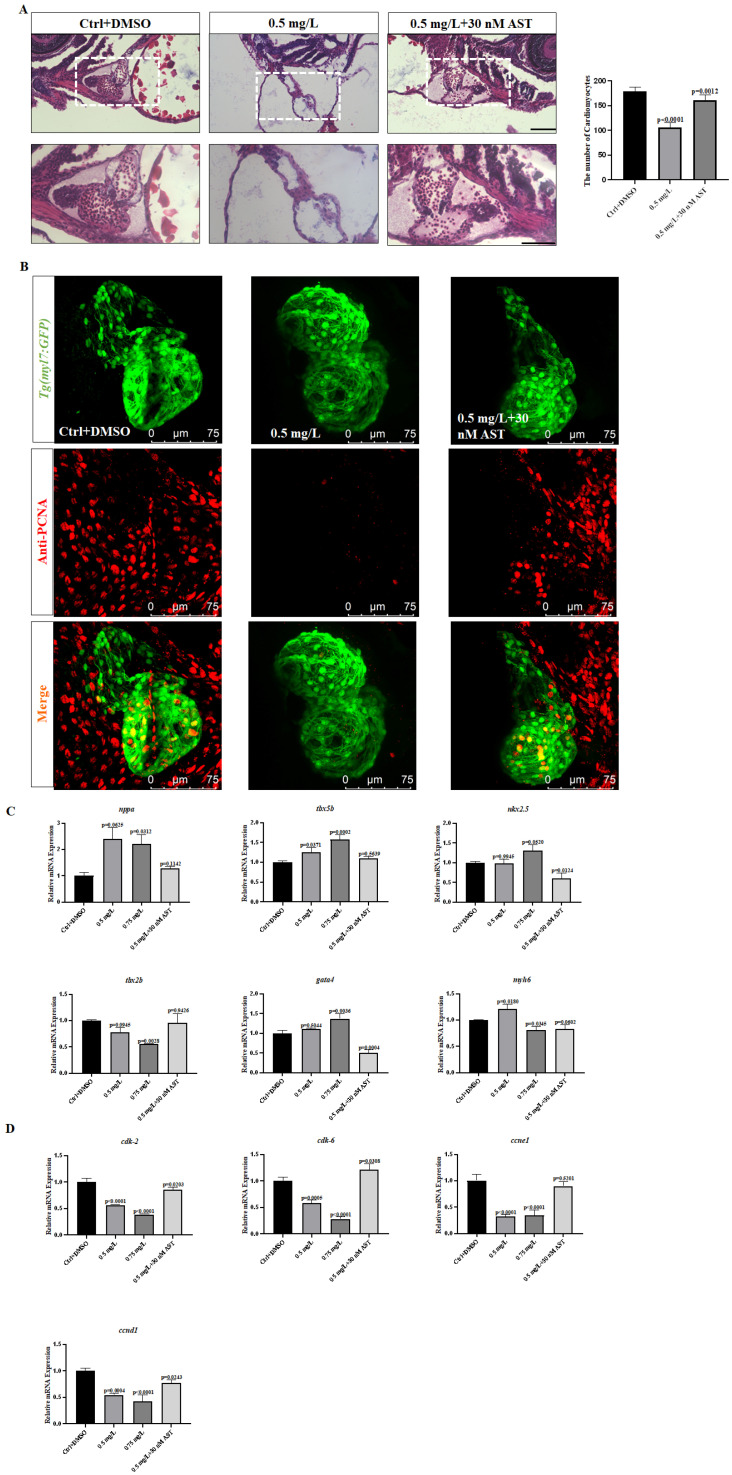
Astaxanthin rescued the expression of genes related to heart and cell proliferation. (**A**). H & E staining images of control group, medium concentration group, and AST rescue medium concentration group. (**B**). The result of immunofluorescence staining after adding AST to rescue. (**C**). AST rescued heart-related gene expression. (**D**). AST rescued cell proliferation related Gene expression status. Each difference is a comparison between the control group and the treatment group. (*n* = 20, Means ± SD. Scale bar: (**A**) 50 μm/25 μm).

Astaxanthin is an antioxidant. We used AST in the rescue medium concentration treatment group to determine whether PCNB caused the toxicity of zebrafish embryos through oxidative stress. We found that it had a significant rescue effect on the body. The degree of lipid peroxidation was reduced (MDA), which reduced the activity of CAT in the body, and the body’s ability to scavenge free radicals (SOD) was improved (Figure 4C,D). Astaxanthin saved the linearization of the heart (Figure 4A and Figure 5A), reducing the level of oxidative stress caused by PCNB (Figure 4B), and increased the expression of heart proliferation-related genes (*cdk-2*, *cdk-6*, *ccnd1*, *ccne1*) (Figure 5D), and the abnormal expression of heart-related genes (*tbx2b*, *tbx5a*, *nppa*, *gata4*) tended to normal expression (Figure 5C). Thus, AST obviously rescued the abnormal cardiac development caused by PCNB in many aspects. The results of immunofluorescence staining showed that, in the group that added AST for rescue, the co-localization of PCNA antibody and cardiomyocytes with green fluorescence increased significantly, indicating that AST can rescue the reduction of cardiomyocyte proliferation (Figure 5B).

## 4. Discussion

Pentachloronitrobenzene is an organochlorine protective fungicide widely used in agriculture for crop production and maintenance. Few reports have focused on the concentration of PCNB in surface water. In 2004, Vincelli [47] et al. simulated by the TurfPQ model, used the half-life of PCNB, the organic carbon content of the lawn, and the daily temperature and precipitation as parameters. The simulated PCNB concentration in the surface water, although the results are often high, can also provide us with some reference. The results show that the concentration of PCNB can reach 0.647 mg/L in surface water. At present, in China and other countries, many reports indicate that PCNB has been detected in herbal medicines and vegetables. In Chinese cabbage, the maximum concentrations of PCNB in roots and leaves reached 112 and 86 ng/g [12]. In aquatic environments, the LC50 for rainbow trout and bluegill sunfish are 0.55 and 0.1 mg/L [10], respectively. Exposure to PCNB has been reported to cause skin and eye irritation in humans [13] and it can also lead to severe oxidative stress in earthworms [48], but the impact of PCNB on cardiac toxicity is unknown. Zebrafish can serve as an excellent model for cardiovascular research [17]. In this article, we have investigated the toxicity, especially cardiotoxicity, of PCNB on zebrafish embryos.

Although there are some reports that the concentration of PCNB in the environment is at the level of ng/L, due to the bioconcentration effect the concentration in the organism is often higher than the concentration detected in the environment. The exposure concentration used in this paper is based on the environmental concentration. The PCNB concentration detected in zebrafish embryos was used as a reference, and the concentration was set on the premise that zebrafish embryos exhibited toxic reactions but not lethality. We selected 0.25 mg/L, 0.5 mg/L and 0.75 mg/L. We found that PCNB was highly cardiotoxic to zebrafish embryos in a concentration-dependent manner. The development of the heart is extremely important for zebrafish survival and requires a complex and orderly process [16,49]. Zebrafish heart development has been reported to be primarily regulated by activation of several genes and transcription factors, such as *tbx5a*, *nppa*, *myh6*, *tbx2b*, and *nkx2.5*. The *nppa* encodes an atrial natriuretic peptide, which can exhibit a diuretic effect and regulate the volume of extracellular fluid and electrolyte homeostasis. Moreover, *nppa* is a chamber-specific marker gene [40]. *Gata4* is an essential cardiac transcription factor whose deletion can cause congenital heart disease [50]. In rats, oxidative stress can induce substantial pathological myocardial injury through promoting up-regulation of *gata4* expression [51]. *Tbx2b* is responsible for regulating the proliferation of cardiomyocytes during early cardiac development [22] and is also actively involved in the regeneration of zebrafish liver cells [52]. In addition, *tbx2b* plays a vital role in the cyclization of the heart [29]. A number of previous studies have shown that both *tbx2a* and *tbx2b* are expressed in the primordial heart tubes of early zebrafish embryos [53], and can effectively promote the proliferation of cardiomyocytes at this stage [22]. The deletion of *tbx2a* and *tbx2b* genes can lead to zebrafish ventricular development defects, which might be related to the decrease of cardiomyocyte proliferation and can hinder the normal loop ability of the heart. Interestingly, the data obtained from Real-time fluorescence quantitative PCR showed that PCNB substantially suppressed the expression of genes associated with cardiac development. The results of real-time quantitative PCR in this study indicated that the expression of *tbx2b* gene was significantly down-regulated, thereby suggesting that the cardiotoxicity of PCNB to zebrafish embryos might be primarily due to the inhibition of the proliferation ability of cardiomyocytes.

To further explore whether PCNB can reduce the proliferative capacity of cardiomyocytes, we performed immunofluorescence staining of zebrafish hearts using PCNA antibody [54]. The result showed that, as the concentration of the drug increased, the number of PCNA antibody-labeled cells that co-localized with *myl7*-labeled cardiomyocytes [55] was significantly reduced, thus implying that the proliferation ability of zebrafish cardiomyocytes was substantially inhibited. We also detected the expression levels of the various proliferation-related genes *cdk-2*, *cdk-6*, *ccnd1*, *ccne1* [34,35]. We found that the levels of proliferation-related genes were significantly reduced. This finding was consistent with the previous reports that the reduced levels of the *tbx2b* gene inhibited cardiac proliferation [22]. We also performed H&E staining of the paraffin sections of the heart and found that the heart was significantly linearized, whereas the number of cardiomyocytes was significantly reduced [19]. This observation suggested that the abnormal zebrafish heart development caused by PCNB might be due to the decreased proliferation of heart cells and substantial reduction in the ability of the heart to loop. There can be several reasons for the reduced ability of cells to proliferate, including aging, oxidative stress [39], etc.

On the contrary, exposure to various harmful external stimuli and pollutants can increase the accumulation of reactive oxygen species in the body, thereby triggering oxidative stress [42,56]. Oxidative stress can lead to increased ROS in the body, causing apoptosis [57], neurotoxicity in zebrafish [37], cardiomyocyte cell cycle arrest [39], etc. In addition, oxidative stress is closely related to many chronic diseases, such as diabetes [58], cardiovascular disorders [59], aging [60], etc., and has received significant attention. According to our results, ROS levels in zebrafish embryos were significantly increased after exposure to PCNB, primarily in the head and heart. The enzyme activity assay showed that SOD was markedly decreased, whereas MDA and CAT were significantly increased. Superoxide dismutase represents the ability of the body to effectively scavenge free radicals [61]. CAT displays the activity of catalase in the body [62], whereas MDA is the product of lipid oxidation, which can reflect the degree of lipid peroxidation in the body and thus indirectly indicate the degree of cell damage [63]. The results of enzyme activity assays showed that PCNB exposure led to substantial reduction of the zebrafish embryo’s ability to scavenge the free radicals, thereby increasing the lipid oxide content in the body, and causing an imbalance between the body’s oxidation and antioxidant levels, i.e., PCNB promoted severe oxidative stress in zebrafish embryos. Cardiotoxicity in zebrafish may thus have occurred due to increased levels of oxidative stress. Since PCNB causes severe oxidative stress, we selected AST, an antioxidant that has been reported to effectively reduce oxidative stress levels by inhibiting cellular production of ROS, which can increase the activity of immune cells and thereby exert a protective effect on the body [40,41]. AST not only has the effect of relieving oxidative stress in cells and mice, but the experimental results of Cao and Huang et al. also proved that AST can play an antioxidant role in zebrafish embryos and can alleviate the cardiotoxicity caused by oxidative stress [18,42]. We used AST to conduct rescue experiments and achieve obvious protective effects. We observed that, in terms of oxidative stress, the expression levels of two SOD-encoding genes, *sod1*, *sod2* [64], were found to be significantly increased. Superoxide dismutase, MAD, CAT appeared to be normal, thus indicating that the level of oxidative stress was significantly reduced. In terms of cardiac-related marker genes, the expression level of *tbx2b* gene was noted to have increased. The expression of proliferation-related genes was significantly up-regulated. Based on the above findings, AST eliminated the cardiotoxicity of PCNB in zebrafish embryos by reducing the level of oxidative stress.

## 5. Conclusions

This paper explored the potential effect of PCNB on zebrafish embryonic development. We found that PCNB caused increased cardiotoxicity and oxidative stress in zebrafish embryos. After exposure to PCNB, the expression levels of the various marker genes linked to the cardiac development in zebrafish embryos were attenuated, and the expression of proliferation-related genes (*cdk-2*, *cdk-6*, *ccnd1*, *ccne1*) was significantly reduced. This indicated that PCNB induced cardiotoxicity by inhibiting the proliferation of zebrafish embryonic cardiomyocytes. The level of PCNB-induced oxidative stress in zebrafish embryos was significantly reduced by exposure to the antioxidant AST. Interestingly, the proliferation level of zebrafish cardiomyocytes was also significantly restored. These findings suggested that AST might reduce the toxicity of PCNB in the heart of zebrafish embryos by significantly inhibiting the level of oxidative stress.

Taken together, PCNB caused serious damage to the zebrafish heart through stimulating oxidative stress, and affected the proliferation of cardiomyocytes, thereby causing cardiotoxicity to zebrafish embryos. As a result, PCNB might function as a more toxic agent, and can exhibit substantial cardiotoxic effects on aquatic organism development or on humans. It might be necessary in future to analyze the cardiotoxicity of PCNB on other model organisms, etc., but currently the first priority is to use alternative fungicides as far as possible to reduce the potential harmful impact of PCNB on aquatic organisms and humans.

## Data Availability

Not applicable.

## Supplementary Materials

The following supporting information can be downloaded at: https://www.mdpi.com/article/10.3390/toxics10060299/s1, Figure S1: The results of actual concentration determination. The retention time was about 3.85 min. Calibration curves were obtained prior to each day of analysis and linearity was confirmed from 0.1 mg/L to 0.8 mg/L (*R*^2^ = 0.9978). All measurements were performed in triplicate. PCNB concentration determination. The linear regressions (*R*^2^ > 0.99) generated from a 5-point calibration standard; Table S1: Sequences of primer pairs used in the real-time quantitative PCR reaction; Table S2: The actual concentrations of PCNB during exposure process (mg/L). The concentration of PCNB exposure were relatively constant and maintained within the range of ±10% of the nominal concentration during the exposure process.

## Abbreviations

Pentachloronitrobenzene (PCNB); Reactive oxygen species (ROS); Hour post-fertilization (hpf); Catalase (CAT); Malondialdehyde (MDA); Superoxide dismutase (SOD); N-phenylthiourea (PTU); National Institutes of Health (NIH); Astaxanthin (AST); Lethal Concentration 50% (LC50); Proliferating Cell Nuclear Antigen (PCNA); Hematoxylin-Eosin staining (H & E).
