# Supplementary material for: Pentachloronitrobenzene Reduces the Proliferative Capacity of Zebrafish Embryonic Cardiomyocytes via Oxidative Stress
Source: Toxics. 2022 Jun 1;10(6):299. doi: 10.3390/toxics10060299 (PMC9231182; doi:10.3390/toxics10060299)
Supplement: Supplementary file 1 [file toxics-10-00299-s001.zip › toxics-1736860-supplementary.pdf]

# Supplementary Materials: Pentachloronitrobenzene Reduces the Proliferative Capacity of Zebrafish Embryonic Cardiomyocytes via Oxidative Stress

**Guoqiang Fan, Tianzhu Shen, Kun Jia, Xiaoping Xiao, Zhanfeng Wu, Fanghua Gong and Huiqiang Lu**

**Table S1.** Sequences of primer pairs used in the real-time quantitative PCR reaction.

| | |
|---|---|
| β-actinQPCR FP | CGAGCAGGAGATGGGAACC |
| β-actinQPCR RP | CAACGGAAACGCTCATTGC |
| Myh6-QPCR fp | CACCAGCAGACACTGGATG |
| Myh6-QPCR rp | GCTCCAAGTCCATTCTGAC |
| Nkx2.5-QPCR fp | TGCTTCAGGCTTTTACGCGA |
| Nkx2.5-QPCR rp | GTCTTCCTGACAACAGCCGA |
| gata4 QPCR FP | GCCGGGATACCGCAACTTAT |
| gata4 QPCR RP | AGTACGGAGCTGTCGAAGTG |
| tbx5a QPCR FP | GCTCCTACCACGAACACACA |
| tbx5a QPCR RP | ATCCTCGCTGGAGGAACTCT |
| nppa QPCR FP | GCTCCTGGTTTGGCAGCAG |
| nppa QPCR RP | GAGCTGCTGCTTCCTCTCGG |
| Tbx2b Qpcr FP | AACTGGCAGAGATGCTGGTC |
| Tbx2b Qpcr RP | ACACCGGTCATTTTGGTGGT |
| sod1 Qpcr FP | TGACCGGCACCGTCTATTTC |
| sod1 Qpcr RP | ATGCAGCCGTTTGTGTTGTC |
| sod2 Qpcr FP | TGTTGGAGGCCATAAAGCGT |
| sod2 Qpcr RP | AGTGGGATGAGACCTGTGGT |
| ccnd1 QPCR FP | CCAGAACCTCACCAACTTCC |
| ccnd1 QPCR RP | TGGTCTCTGTGGAGATGTGC |
| ccne1 QPCR FP | CGCAGTATGCATCAGAAAGC |
| ccne1 QPCR RP | TCCATAACGCGTGTATCTCG |
| cdk2 QPCR FP | GATCGGAGAGGGAACATACG |
| cdk2 QPCR RP | GCAGAGAGATCTCACGAATGG |
| cdk6 QPCR FP | GGTGCAGACTGAGGAAGAGG |
| cdk6 QPCR RP | TCCTGGAAACTGTGCATACG |

**Table S2.** The actual concentrations of PCNB during exposure process (mg/L). The concentration of PCNB exposure were relatively constant and maintained within the range of ± 10% of the nominal concentration during the exposure process.

| Concentration (mg/L) | 6 hpf | 24 hpf |
|---|---|---|
| DMSO | ND | ND |
| 0.25 | 0.2482±0.0002 | 0.2483±0.0005 |
| 0.5 | 0.5059±0.0002 | 0.5059±0.0002 |
| 0.75 | 0.7633±0.0002 | 0.7633±0.0001 |

ND: Not detected, <LOD. The data were expressed as mean±standard deviation (SD). *n*=3.

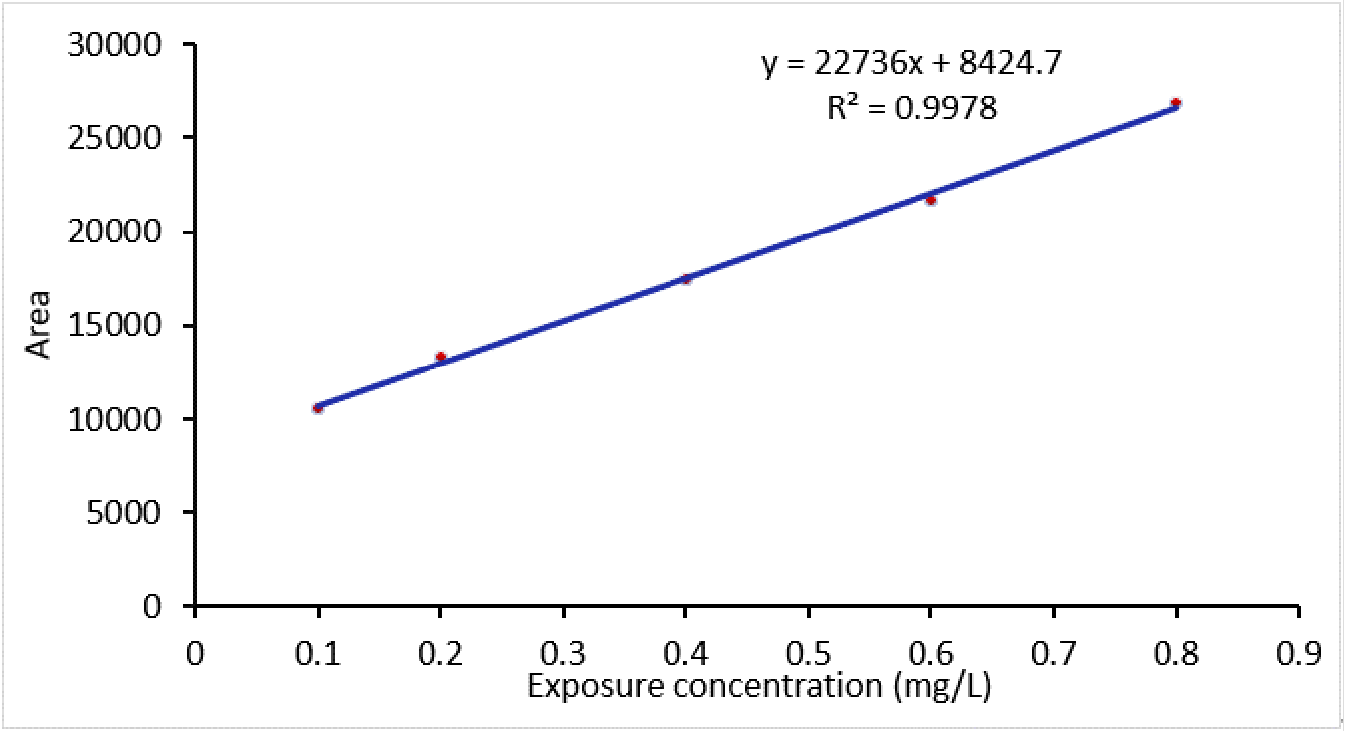


**Figure S1.** The results of actual concentration determination. The retention time was about 3.85 min. Calibration curves were obtained prior to each day of analysis and linearity was confirmed from 0.1 mg/L to 0.8 mg/L ($R^2$ = 0.9978). All measurements were performed in triplicate. PCNB concentration determination. The linear regressions ($R^2$ > 0.99) generated from a 5-point calibration standard.
